# Pathways to prevention: protocol for the CAP (Climate and Preventure) study to evaluate the long-term effectiveness of school-based universal, selective and combined alcohol misuse prevention into early adulthood

**DOI:** 10.1186/s12889-018-5554-y

**Published:** 2018-05-21

**Authors:** Nicola C. Newton, Lexine Stapinski, Tim Slade, Katrina E. Champion, Emma L. Barrett, Catherine Chapman, Anna Smout, Siobhan Lawler, Marius Mather, Natalie Castellanos-Ryan, Patricia J. Conrod, Maree Teesson

**Affiliations:** 1 NHMRC Centre of Research Excellence in Mental Health and Substance Use (CREMS) National Drug and Alcohol Research Centre, University of New South Wales, Sydney, Australia; 2 Feinberg School of Medicine, Northwestern University, Chicago, USA; 3 University of Montreal, Montreal, Canada

**Keywords:** Alcohol, Prevention, Universal, Selective, Combined, School, Personality

## Abstract

**Background:**

Alcohol use and associated harms are among the leading causes of burden of disease among young people, highlighting the need for effective prevention. The Climate and Preventure (CAP) study was the first trial of a combined universal and selective school-based approach to preventing alcohol misuse among adolescents. Initial results indicate that universal, selective and combined prevention were all effective in delaying the uptake of alcohol use and binge drinking for up to 3 years following the interventions. However, little is known about the sustainability of prevention effects across the transition to early adulthood, a period of increased exposure to alcohol and other drug use. This paper describes the protocol for the CAP long-term follow-up study which will determine the effectiveness of universal, selective and combined alcohol misuse prevention up to 7 years post intervention, and across the transition from adolescence into early adulthood.

**Methods:**

A cluster randomized controlled trial was conducted between 2012 and 2015 with 2190 students (mean age: 13.3 yrs) from 26 Australian high schools. Participants were randomized to receive one of four conditions; universal prevention for all students (*Climate*); selective prevention for high-risk students (*Preventure*); combined universal and selective prevention (*Climate and Preventure*; CAP); or health education as usual (Control). The positive effect of the interventions on alcohol use at 12-, 24- and 36-month post baseline have previously been reported. This study will follow up the CAP study cohort approximately 5- and 7-years post baseline. The primary outcome will be alcohol use and related harms. Secondary outcomes will be cannabis use, alcohol and other drug harms including violent behavior, and mental health symptomatology. Analyses will be conducted using multi-level, mixed effects models within an intention-to-treat framework.

**Discussion:**

This study will provide the first ever evaluation of the long-term effectiveness of combining universal and selective approaches to alcohol prevention and will examine the durability of intervention effects into the longer-term, over a 7-year period from adolescence to early adulthood.

**Trial registration:**

This trial was registered in the Australian New Zealand Clinical Trials Registry (ACTRN12612000026820) on January 6th 2012.

## Background

Young adulthood in Australia is chracterized by increased use of alcohol and other drugs [1, 2]. Within a 12 month period, almost one in three young Australian adults consume alcohol at very high risk levels (over 10 standard drinks on one occasion), and 28% will try an illicit drug [3]. At this age, episodes of intoxication can have significant health, legal, social and financial consequences, including risk of impaired neurocognitive function and development [2]. In addition, patterns of alcohol and other drug use established during early adulthood increase risk of developing substance use disorders and co-morbid mental health problems, all of which negatively impact on current functioning and future life options [2]. Among young people aged 20–24, alcohol and other drug use are among the leading contributors to total burden of death, disease and injury, accounting for an estimated 14% of total disease burden [4]. The substantial harm related to alcohol and other drug use is in part attributable to the associated occurrence of violent behaviour, including assaults, homicides, self-harm and suicide [2]. For example, toxicology reports from a national coronial inquiry in Australia found that alcohol and/or drug (primarily cannabis) use was involved in 78% of “King hit” (single incapacitating blow to the head) cases between 2000 and 2012 [5]. Despite the considerable harms attributable to alcohol and other drug use, our prevention evidence base in this age group is weak.

To reduce the occurrence and cost of harms relating to the use of alcohol and other drugs, prevention is essential and needs to be initiated before patterns of use are established and begin to cause harm [6, 7]. Onset of alcohol and other drug use typically occurs during adolescence, and thus secondary schools are an ideal location for prevention given the potential for broad reach while keeping costs low. School-based prevention programs are an effective way to reduce the onset and escalation of alcohol and other drug use [8, 9]. The two most common approaches to substance use prevention are ‘universal’ and ‘selective’ prevention [10]. Universal prevention aims to deliver interventions to all students regardless of their level of risk, predominately focusing focusses on skill development and normative education. Selective prevention on the other hand, involves targeting programs to specific populations, such as individuals at greatest risk for developing problems with alcohol or other drugs. Although both approaches have been found to be effective in preventing substance use among adolescents [11–14], until recently, there were no models which combined both universal and selective approaches to prevention.

### The CAP study: First randomized controlled trial of selective and universal prevention

The *Climate* and *Preventure* (CAP) study was initiated in 2011 as the first randomized controlled trial of a comprehensive prevention approach combining both universal (*Climate Schools* program; delivered to all students) and selective (*Preventure*; tailored to high-risk students) intervention programs. The primary aim of the study was to assess the effectiveness of universal, selective and combined approaches to preventing alcohol use and related harms among adolescents. Twenty-six schools and 2190 Grade 8 students participated in the trial. Results supported the effectiveness of all programs, however indicated no advantage of combining the two interventions (See Table 1). Both the *Climate* program alone or in combination with *Preventure* significantly increased knowledge of alcohol and cannabis-related harms, and lowered the growth in alcohol use and incidence of binge-drinking compared to the control condition [15, 16]. The selective *Preventure* program was also effective among adolescents classified as high-risk on personality dimensions that increase vulnerability to alcohol and other drug use and related-harms. Specifically, among high-risk students who received *Preventure*, growth in alcohol use, binge-drinking and alcohol-related harms was significantly lower control group 3 years following the intervention [17]. These findings replicate results from the UK and Canadian trials, and indicate successful adaptation of the *Preventure* program for students in Australia. At 24-month follow-up, effect sizes for the Climate, Preventure and combined interventions compared to control ranged from d = − 0.21 to d = − 0.56, and Number Needed to Treat (NNT) values ranged from 6 to 12 (see Table 1). These effect sizes and NNTs compare favourably to other alcohol prevention programmes [8].

**Table 1:** Effect sizes and number needed to treat for changes relative to control group in any drinking, binge drinking and alcohol-related harms. Effect sizes are based on Intervention x Time coefficients from latent growth models used to analyze intervention outcomes [16, 58], converting the estimated odds ratios to standardised mean differences d [59]

Participants	Outcome	Intervention group	*b*	OR	d	NNT
All participants *(at 24 months post baseline)*	Any drinking	Climate^*^	−0.38	0.47	−0.42	7
		Preventure^*^	−0.36	0.49	−0.40	7
		CAP^*^	−0.19	0.68	−0.21	12
	Binge drinking	Climate^*^	−0.51	0.36	−0.56	9
		Preventure^*^	−0.41	0.44	−0.45	10
		CAP^*^	−0.36	0.49	−0.40	11
	Alcohol-related harms	Climate	−0.23	0.63	−0.25	10
		Preventure^*^	−0.38	0.47	−0.42	6
		CAP	−0.19	0.68	−0.21	12
High-risk participants *(at 36 months post baseline)*	Any drinking	Preventure^*^	−0.225	0.51	−0.37	7
	Binge drinking	Preventure^*^	−0.305	0.40	−0.50	6
	Alcohol-related harms	Preventure^*^	−0.255	0.47	−0.42	6

^*^: significant at *p* < 0.05

*b*: Intervention x Time coefficient

OR: Estimated odds ratio

d: Estimated effect size (standardised mean difference)

NNT: Number needed to treat to benefit (based on OR and the event rate in the control group)

### The need for longer-term follow-up over the transition to early adulthood

The CAP trial cohort is now approaching early adulthood, a period of increased use of alcohol and other drugs, and heightened risk of harms associated with this use including injury, self-harm, violent behaviour, and onset of substance use disorders [1, 2]. Adolescents with personality risk factors for alcohol and other drug use, such as impulsiveness and sensation-seeking [18, 19], may be particularly susceptible during this period of new found autonomy. This transition also encompasses legal access to alcohol at 18 years in Australia. Increasingly regular drinking and other drug use may emerge as a way of forming new friendships, or to cope with the increasing life stressors and demands that emerge at this stage. Previous investigations by our team highlight the importance of coping motives for alcohol use (i.e. drinking to cope with negative emotions such as anxiety or hopelessness), which are strongly associated with alcohol-related harms during this early period of adulthood [17].

Recent results from the Australian National Drug Strategy Household Survey show that the average age of initiation for alcohol use and other substances such as cannabis has increased among young Australians [3]. This delayed initiation may mean that some effects of preventative interventions do not emerge until later, after participants’ peers have begun to use, and exposure to alcohol and other substances increases. This underscores the need to assess long-term effects of the interventions to see how they affect use and harms during this critical period.

Despite the unique challenges and increased susceptibility during young adulthood, few studies have examined the effectiveness of substance use prevention beyond secondary school. Recent results from a study examining the effects of universal prevention implemented in adolescence showed benefits sustained into early adulthood, whereby the intervention group reported significantly lower levels of substance misuse 7.5 years past baseline across a range of substances [20]. Similarly, studies evaluating the long-term outcomes of universal skills training implemented in early adolescence indicate reduced risk of alcohol-related problems and illicit drug use into early adulthood (ages 18–22) [21–23].

The long-term effectiveness of selective prevention approaches (such as *Preventure*), and the combination of skills development within a social influence model (such as *Climate*) is unknown. The durability of universal and selective prevention effects into early adulthood is a crucial research question, as the choices young people make during this important life stage can have profound effects for many years to come.

#### Secondary effects of alcohol and drug use: Violence and aggression

The increased risk of aggression and violent behaviour among young adults who misuse alcohol or other drugs is of significant concern [24]. In a survey of over 8000 Australian students, those who engaged in binge drinking were 5 times more likely to be violent (i.e. attack someone to serious hurt or injure them) compared to non-binge drinkers [25]. Moreover, the probability of having committed a serious violent offence increases from 9 to 79% between the ages of 14 to 20 among male adolescents with a history of alcohol use disorder [26]. Previous research indicates that the relationship between these behaviours is complex and further investigation is required in an Australian sample to better understand how alcohol, illicit drugs and aggression and violent behaviour interact during the transition from early adolescence to young adulthood [27]. Research has identified specific substance use-related personality risk factors (i.e. impulsivity and hopelessness) as important targets for interventions aimed at reducing aggression and delinquent behaviour [28]. Interventions that effectively prevent alcohol and other drug use hold promise for reducing associated aggression and propensity for violence, however these potential benefits have not yet been examined.

### The CAP long-term follow-up study

The CAP *long-term follow up* is an opportunistic extension of the CAP study, whereby the cohort will be assessed at two additional times (5- and 7-years post baseline) over the critical transition period into early adulthood. There is limited evidence about the sustainability of prevention effects beyond 3 years, and this study will provide important information about which prevention approaches are most sustainable. The primary objective of the study is to assess the long-term effectiveness of universal, selective and combined prevention approaches to reducing alcohol use and related harms compared to control (drug education as usual) at approximately 5 and 7 years post intervention. Primary outcomes are: frequency of alcohol use, frequency of binge drinking (consuming 5 or more standard drinks on a single occasion), alcohol-related harms and onset of an alcohol use disorder. A second objective is to assess the effectiveness of the prevention approaches in reducing cannabis use, incidence of cannabis use disorder, mental health symptoms, and in reducing secondary harms associated with alcohol use, specifically aggression and violence during the high-risk early adulthood period.

## Methods/Design

### Study design

**Fig. 1:**
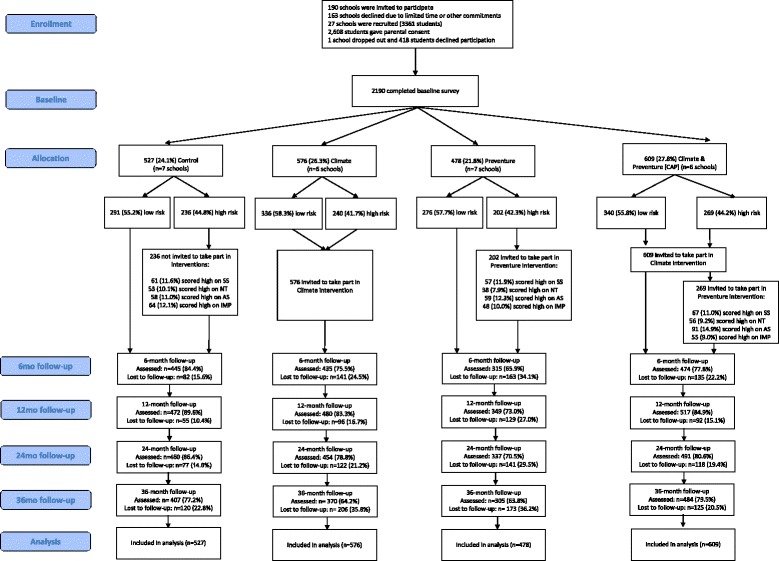
CONSORT figure for participant flow in the CAP Trial

A total of 27 schools (*n* = 2260) were recruited to the CAP trial in 2012. One school (assigned to the Climate condition) withdrew after randomization (due to time constraints), but prior to completing baseline questionnaires. The final cohort at baseline consisted of 2190 Year 8 students from 26 schools (mean age = 13.3 years, 57.4% male, 86% born in Australia). All participants were screened at baseline for personality risk-factors (Sensation Seeking, Impulsivity, Anxiety Sensitivity and Hopelessness) using the Substance Use Risk Profile Scale (SURPS) [29]. Consistent with previous research from the United Kingdom [19, 30], 43% of the sample were identified as high-risk (> one standard deviation above the school mean) on one of the four personality dimensions. Participating schools were randomized by an external researcher using the online program Research Randomizer (www.randomiser.org) to one of four conditions: (1) Control (health education as usual), (2) Climate (universal prevention for all), (3) Preventure (selective prevention delivered to students with high-risk personality profiles), or (4) Climate and Preventure (both universal and selective approaches). Blocked randomisation was used, allocating schools to the four conditions in equal ratios in blocks of 4. The CONSORT diagram (see Fig. 1) summarizes participant flow and retention rates through the study for each condition. Comprehensive information about the intervention content and delivery has been published previously [15–17, 31], and the full study design of the original CAP trial has been published in the original CAP study protocol [31]. The completed CAP study assessments and timeline for extended follow-up assessments can be seen in Table 2. The CAP long-term follow-up trial will extend data collection up to 7 years post baseline. Using multiple sources of locator information already provided to the research team, all participants will be invited to complete two online assessments at approximately 5- and 7- years post baseline. Participants will complete surveys online via the CAP study website (www.capstudy.org.au), and their responses will be linked over time using their unique identification code.

**Table 2:** Completed CAP study assessments and timeline for extended follow-up assessments

Original CAP trial					Timeline for Extended Follow-up	
2012	2012	2013	2014	2015	2017-2018	2019
Year 8	Year 8	Year 9	Year 10	Year 11	Post-school +1	Post-school +3
Baseline + Intervention	6/9-mth F/U survey	1-year F/U survey	2-year F/U survey	3-year F/U survey	5-year F/U survey	7-year F/U survey
*n* = 2,190	*n* = 1,669 (76%)	*n* = 1,818 (83%)	*n* =1,732 (79%)	*n* =1,601 (71%)		

### Sample size calculations

Power calculations for the original trial were based on recent methods developed to account for cluster randomisation [32]. These calculations indicated that 600 high risk students from 20 schools would achieve 80% power to detect a between-group standardized mean difference of 0.30 (*p* < 0.05) for comparisons among high risk students. Since high-risk students were expected to represent 40% of participants, this meant 1500 students would be required in total. Allowing for 20% dropout, the final required sample size was 1920 students from 24 schools. In our original study, we achieved well over this recruitment target, with 2190 students and 947 high-risk students participating in the study.

As initiation and frequency of alcohol and cannabis use increase over the transition to early adulthood [1, 33], power to detect changes in use should increase due to the greater prevalence. Previous prevention research has also shown larger effect sizes with longer-term follow-up, so our original expected effect sizes may be conservative [8, 22]. Thus, even allowing conservatively for drop-out rates of > 35%, the study should remain adequately powered for the expected size of effect for Climate (0.2) and Preventure (0.3) – calculations of power using the original methods and the obtained sample size show that the study has > 80% power to detect effects of these sizes at the long-term follow-up occasions.

### Cohort analysis

The cohort for the long-term follow up study comprises students from the 26 schools that were recruited to the original CAP study in 2012. Of the participating schools, there were nine government, six Catholic, and 11 independent schools from New South Wales (NSW), with one independent school from Victoria (VIC). These included 10 boy’s schools, nine girl’s schools and eight co-educational schools. In 2012, at the outset of the trial, the included schools had a median ICSEA (Index of Community Socio-Educational Advantage) score of 1131 (interquartile range 1048 to 1155). ICSEA is an index of the average educational advantage of the students in Australian schools based on factors including parent education and occupation, where higher scores represent more advantage and 1000 is the average of all Australian schools [34]. Consent forms were sent home to parents of all Year 8 students at participating schools. Independent schools (*n* = 17) used passive parental consent procedures, while public schools (*n* = 9) required active parental consent to satisfy ethical requirements. Any student who was Year 8 in 2012 and provided parental consent was eligible to participate.

Of the baseline cohort (*n* = 2190), 527 students (7 schools; 24.1%) were randomly allocated to the Control group, 576 students (6 schools; 26.3%) to the Climate group, 478 students (7 schools; 21.8%) to the Preventure group, and 609 students (6 schools; 27.8%) to the Climate & Preventure group. There were some differences at baseline between students at the intervention and control schools. There was a greater distribution of males in schools allocated to the Preventure (82%) and CAP (79.3%) condition compared to the Climate (35.9%) and Control (33.1%) schools (χ^2^_(3)_ = 472.793, *p* < 0.01). Figure 1 summarises the distribution of low- and high-risk students in each intervention condition. Low risk students were those who did not meet personality risk criteria (i.e., 56.8% of the Year 8 population).

No information about non-participants was gathered, as contact details were not obtained prior to baseline survey completion. However, socio-economic and demographic indicators suggest the sample is nationally representative. The proportion of the sample born in Australia (86%) is comparable to Australian population statistics (71.8%) [35], and the schools’ Index of Community Socio-Educational Advantage was marginally above the Australian average.

Student follow-up in the original CAP trial occurred annually. To date, five waves of survey data have been collected; baseline, post-intervention (approximately 6–9 months post baseline), 1-, 2- and 3-year follow-ups. This study will extend assessment of the cohort, conducting two post-school follow-ups (5- and 7-years post-baseline). Over the first five waves of data collected, retention was high ranging from 71.5 to 83% (See Table 2). The most common reasons for attrition were students being absent on the day of the survey, failing to use their correct unique identification code or answering fewer than 80% of questionnaire items within any scale. Only a small number of students (*n* = 114, 5.2%) completed baseline assessment only; these students consumed alcohol more frequently (F _(1, 2180)_ = 11.04, *p* < 0.01), and in greater quantities (F _(1, 2180)_ = 20.45, *p* < 0.01), had significantly higher binge drinking (F _(1, 2187)_ = 11.12, *p* < 0.01) and more alcohol-related harms (F _(1, 2187)_ = 20.53, *p* < 0.01) compared to students who completed at least one follow-up assessment. It should be mentioned that evidence of differential attrition was observed between the control and intervention conditions on the outcome measures over a 2-year follow-up assessment. Specifically, attrition was more likely to occur in the Preventure group compared to the other groups (See Fig. 1).

All participants who completed baseline assessments remain eligible to participate in follow-up assessments and will continue to be contacted, meaning participants absent at previous follow-ups can still be assessed. As discussed below, in order to account for missing data due to attrition, maximum likelihood estimation methods will be used to handle missing data in all analyses of trial outcomes.

### Measures

Demographic data including gender, age, academic performance, and truancy rates were collected at baseline. To allow for modelling of outcomes over a 7-year period, the extended follow-up assessments will use the same primary outcome measures as the original trial. All assessments will involve self-report using well-validated instruments. Self-report is the favoured method of assessment for young people for measuring drug and alcohol use and related harms and has been found to have excellent discriminant and predictive validity [36].

#### Primary outcomes

Drinking and binge drinking will be assessed using an adapted version of the Patterns of Alcohol index [37] which will facilitate comparison with a large scale representative group of Australians. Drinking frequency will be measured using an item asking how often in the past 6 months participants have consumed a standard drink of alcohol, with responses ranging from “Never” to “Daily or almost daily”. Binge drinking frequency will be assessed using a similar item asking how often participants consumed 5 or more standard drinks on a single occasion. Alcohol-related harms will be measured using an abbreviated version of the Rutgers Alcohol Problem Index [38] and the DSM-5 self-report symptom checklist developed by Batterham, Sunderland [39] will be administered to assess emerging symptoms of alcohol use disorder.

#### Secondary outcomes

Cannabis use will be assessed using cannabis questions from the Australian National Drug Strategy Household Survey 2013 [33], which also allows for comparison with a large scale representative group of Australians. A single item with Yes/No responses asks participants whether they have used cannabis in the past 6 months. Aggression will be measured using the Reactive-Proactive Aggression Questionnaire [40] using the combined total of the reactive and proactive aggression scales, as well as the adult version of the Strengths and Difficulties Questionnaire (SDQ18+) [41] . Self-reported violence will be measured using an adapted version of the Self-Reported Delinquency scale, including 7 items related to violent behaviours. In line with previous research utilising the scale and the serious nature of the events specified, individuals who report one or more violent acts in the past year will be defined as self-reported violent offenders. The original version of the scale has been validated and used in the Dunedin Multidisciplinary Health and Development Study [42, 43].

Other measures that will be administered include the following: i) the Drinking Motives Questionnaire-Revised [44] to assess drinking motives across four dimensions (social, conformity, enhancement, and coping motives) [44]; ii) a 5 item perceived peer use scale will assess participants’ estimation of the proportion of their friends who use alcohol and cannabis [31]; iii) the Kessler-6 [45] and the adult version of the Strengths and Difficulties Questionnaire (SDQ18+) [41] will assess emotional and behavioural problems; iv) Specific depression and anxiety symptoms will be measured by relevant subscales of the Brief Symptom Inventory [46]; v) the Child Health Utility Instrument (CHU-9D) [47], a preference-based health-related quality of life measurement, will be used in order to quantify the cost effectiveness of the intervention in combination with items taken from the Young Minds Matter service utilization module [48]; vi) two items validated for use among adolescents will assess whether participants’ responses are truthful [49–51].

### Statistical analysis

#### Modelling approach

Primary and secondary outcomes will be analyzed in longitudinal analyses using multilevel mixed effects regression models. The effects of greatest interest are Intervention × Time interactions that reflect differences between intervention groups in the growth of each outcome over time. Multilevel modelling can account for the expected correlations between different observations of the same individual and between individuals in the same school [52], which would otherwise violate assumptions of independence in traditional regression models. Therefore, models used in these analyses will incorporate both random intercepts and slopes for time at the individual level, and random intercepts at the school level. Mixed effects regression approaches that use maximum likelihood estimation are superior to alternative missing data strategies such as pairwise deletion [53]. Maximum likelihood methods produce unbiased estimates when missing data is assumed to be either missing completely at random (MCAR) or missing at random (MAR) [54].

Generalized linear modelling approaches will be applied where appropriate, so that logistic regression with a logit link function will be applied when analysing binary outcomes. To determine the best fitting model for each outcome, possible fixed effects and random effects structures will be compared using likelihood ratio tests and model fit statistics such as the Akaike information criterion. For all outcomes, measures of effect size such as standardized mean differences (e.g. Cohen’s *d*) and odds ratios will be calculated along with their corresponding 95% confidence intervals to provide interpretable estimates of the intervention effects. All analyses will be carried out on an intention-to-treat basis, retaining and analysing all participants in the intervention groups they were originally allocated to.

#### Planned comparisons

The primary aims of the original CAP trial were to assess the efficacy of the combined CAP intervention in comparison with the stand-alone *Climate* intervention and the standard health education received by the control group [31]. Therefore, planned comparisons for each outcome will compare CAP *v.* Control, Climate *v.* Control, and CAP *v.* Climate, including all participants allocated to each of these intervention groups.

For high-risk participants, the aim is to compare the efficacy of the *Preventure* program with that of the standard health education received by the control group. Planned comparisons will compare Preventure *v.* Control, including all participants allocated to these intervention groups who were classified as high risk at baseline.

#### Procedure

All students who did not withdraw their consent to participate in the original CAP study will be contacted via email from July 2017, inviting them to participate in the long-term follow up study. A personalized URL will lead participants directly to the CAP survey site where they can complete written consent procedures followed by the first survey (approximately 40–60 min in duration) online. Participants use unique login details, allowing their data to be linked over time through their unique identifier code. First contact with students will be made via email invitation, with reminder emails and texts sent once a week for 3 weeks. Those who cannot be reached via email will be contacted via alternative forms of locator information, including SMS and social media (Facebook). If these attempts are unsuccessful in generating a response, participants will be phoned and paper surveys will be mailed to their home address. Schools will also be contacted in the first week with the option of circulating a flyer, or sending a link to the survey to their participating alumni student contacts. Participants will be reimbursed $30 for each survey occasion they complete. Additionally, all participants who complete each survey will be eligible to win an iPhone, with winners chosen by random selection.

## Discussion

This paper describes the design and protocol of an extended longitudinal follow-up of the CAP study cohort. This study will evaluate the long-term effectiveness of universal, selective and combined approaches to prevent alcohol misuse and related harms among Australian youth as they transition into early adulthood. The effectiveness of the *Climate*, *Preventure* and combined CAP program in reducing alcohol use, binge-drinking and related harms will be assessed relative to health and drug education as usual at approximately 5- and 7-years post baseline. In addition, we aim to ascertain whether selective and universal prevention approaches are effective in reducing cannabis use, mental health symptoms, and aggression and violence into early adulthood, in comparison to education as usual.

### Strengths and limitations

This study will examine the durability of universal and selective prevention programs over a critical 7-year period from adolescence to early adulthood, addressing a crucial knowledge gap and indicating which prevention approaches are most sustainable long-term. Moreover, this study is the first to examine whether combining universal and selective prevention strategies enhances durability of effects in the longer-term. As with the original CAP study, a limitation of this study is the reliance on self-report. Adolescents are reporting on their risky or illegal behaviours, thus their responses may be subject to social desirability bias. Nonetheless, studies have advocated for the excellent discriminant [55] and predictive [36] validity of self-report measures in the assessment of substance-related symptoms in young people [30, 56]. Two validity screening items validated across three studies [49–51] have also been added to this questionnaire in an attempt to discern the proportion of untruthful responders, in addition to the existing techniques utilized to maximize the accuracy of self-reports (e.g. visual prompts, paper and pencil assessments). Data attrition is another potential limitation to this study, with higher levels of attrition predicted over the 7-year follow up due to students being out of school and more difficult to reach. Students providing incorrect or incomplete contact details, being overseas, or being less inclined to take part without the encouragement of teachers, parents and researchers are all anticipated barriers. Financial reimbursement with regular and varied reminders have been associated with high retention rates in clinical trials [57]. To encourage participation, the participants will be reimbursed $30 for survey completion, and will be eligible to win an iPhone. Participants will receive regular prompts via email, text message and phone call.

### Implications

Despite the substantial harms attributable to alcohol and drug use, few studies have examined the effectiveness of prevention strategies beyond secondary school in preventing substance use and related harms. Thus, little is currently known about the durability of universal, selective and combined preventive intervention efforts beyond 3 years. This paper describes the study protocol, design and current implementation of an extended cluster randomized controlled trial to evaluate the long-term effectiveness of the CAP study, the first comprehensive prevention initiative combining both selective and universal intervention components. The extension of follow-up to 7-years post baseline will provide valuable insight into what type of program is most effective and efficient in preventing alcohol use and related harms into early adulthood, a critical developmental period. It will also determine any benefit of these interventions for preventing cannabis use over this period of increasing cannabis exposure. Moreover, it will determine the effectiveness of selective and universal prevention approaches in reducing harms associated with alcohol, including aggression and violence, into young adulthood.

This knowledge is vital to inform policy nationally and internationally, as economic modelling suggests substantial societal benefit can be gained from even modest reductions in drug and alcohol use (38). Evidence of sustained benefits into adulthood would provide an existing and scalable prevention strategy that could be disseminated immediately, at minimal cost, to reduce the considerable harms of alcohol misuse among young Australians.

## References

[1] Teesson M (2010). Prevalence and correlates of DSM-IV alcohol abuse and dependence in Australia: findings of the 2007 National Survey of mental health and wellbeing. Addiction.

[2] Hall WD, et al. Why young people’s substance use matters for global health. Lancet Psychiatry. 2016;3(3):265–79. 10.1016/S2215-0366(16)00013-4 26905482

[3] Australian Institute of Health and Welfare (2017). National Drug Strategy Household Survey (NDSHS) 2016 key findings.

[4] Degenhardt L (2016). The increasing global health priority of substance use in young people. Lancet Psychiatry.

[5] Pilgrim JL, Gerostamoulos D, Drummer OH (2014). “King hit” fatalities in Australia, 2000–2012: the role of alcohol and other drugs. Drug Alcohol Depend.

[6] Spooner C, Hall W (2002). Public policy and the prevention of substance-use disorders. Curr Opin Psychiatr.

[7] Botvin GJ, Griffin KW (2007). School-based programmes to prevent alcohol, tobacco and other drug use. Int Rev Psychiatry.

[8] Foxcroft DR, Tsertsvadze A. Universal school-based prevention programs for alcohol misuse in young people. *Cochrane Database of Systematic Reviews* 2011, Issue 5. Art. No.: CD009113. 10.1002/14651858.CD009113. 10.1002/14651858.CD009113 21563171

[9] Teesson M, Newton NC, Barrett EL (2012). Australian school-based prevention programs for alcohol and other drugs: a systematic review. Drug Alcohol Rev.

[10] Foxcroft DR (2014). Can prevention classification be improved by considering the function of prevention?. Prev Sci.

[11] Stockings E (2016). Prevention, early intervention, harm reduction, and treatment of substance use in young people. Lancet Psychiatry.

[12] Foxcroft DR, Tsertsvadze A (2012). Cochrane review: universal school-based prevention programs for alcohol misuse in young people. Evidence Based Child Health Cochrane Rev J.

[13] Conrod P (2016). Personality-targeted intervenions for substance use and misuse. Curr Addict Rep.

[14] Lee NK, et al. What works in school-based alcohol education: a systematic review. Health Educ J. 2016;75(7):780–98.

[15] Champion KE (2016). A cross-validation trial of an internet-based prevention program for alcohol and cannabis: preliminary results from a cluster randomised controlled trial. Aust N Z J Psychiatry.

[16] Teesson M (2017). Combined universal and selective prevention for adolescent alcohol use: a cluster randomized controlled trial. Psychol Med..

[17] Stapinski LA (2016). Drinking to cope: a latent class analysis of coping motives for alcohol use in a large cohort of adolescents. Prev Sci.

[18] Newton N (2016). Validity of the substance use risk profile scale in Australian adolescents. Addict Behav.

[19] Castellanos-Ryan N, Conrod P, Verster C (2012). Personality and substance misuse evidence for a four-factor model of vulnerability. Drug abuse and addiction in medical illness: causes, consequences and treatment.

[20] Spoth R (2017). PROSPER delivery of universal preventive interventions with young adolescents: long-term effects on emerging adult substance misuse and associated risk behaviors. Psychol Med..

[21] Spoth RL (2006). Long-term effects of universal preventive interventions on methamphetamine use among adolescents. Arch Pediatr Adolesc Med.

[22] Spoth R (2014). Replication RCT of early universal prevention effects on young adult substance misuse. J Consult Clin Psychol.

[23] Ellickson PL, Bell RM, McGuigan K (1993). Preventing adolescent drug use: long-term results of a junior high program. Am J Public Health.

[24] Miller PG (2016). Relationships between problematic alcohol consumption and delinquent behaviour from adolescence to young adulthood. Drug Alcohol Rev.

[25] Williams J (2009). Violent and antisocial behaviours among young adolescents in Australian communities: an analysis of risk and protective factors.

[26] Plattner B (2012). Psychopathology and offense types in detained male juveniles. Psychiatry Res.

[27] Moore SE (2014). Adolescent peer aggression and its association with mental health and substance use in an Australian cohort. J Adolesc.

[28] Castellanos-Ryan N, Conrod PJ (2011). Personality correlates of the common and unique variance across conduct disorder and substance misuse symptoms in adolescence. J Abnorm Child Psychol.

[29] Woicik P (2009). The substance use risk profile scale: measuring traits linked to reinforcement-specific substance use profiles. Addict Behav.

[30] Conrod PJ, Castellanos N, Mackie C (2008). Personality-targeted interventions delay the growth of adolescent drinking and binge drinking. J Child Psychol Psychiatry.

[31] Newton NC (2012). The CAP study, evaluation of integrated universal and selective prevention strategies for youth alcohol misuse: study protocol of a cluster randomized controlled trial. BMC Psychiatry.

[32] Heo M, Leon AC (2009). Sample size requirements to detect an intervention by time interaction in longitudinal cluster randomized clinical trials. Stat Med.

[33] AIHW (2014). 2013 National Drug Strategy Household Survey report, in Drug statistics series no. 28. Cat. no. PHE 183.

[34] Australian Curriculum Assessment and Reporting Authority, Guide to understanding ICSEA. 2012, Australian Curriculum Assessment and Reporting Authority.

[35] Australian Bureau of Statistics. 3412.0 - Migration, Australia, 2015–16. Canberra; 2017.

[36] Crowley TJ (2001). Validity of structured clinical evaluations in adolescents with conduct and substance problems. J Am Acad Child Adolesc Psychiatry.

[37] McBride N (2004). Harm minimization in school drug education: final results of the school health and alcohol harm reduction project (SHAHRP). Addiction.

[38] White HR, Labouvie EW (1989). Towards the assessment of adolescent problem drinking. J Stud Alcohol.

[39] Batterham PJ (2015). Developing a roadmap for the translation of e-mental health services for depression. Aust N Z J Psychiatry.

[40] Raine A (2006). The reactive–proactive aggression questionnaire: differential correlates of reactive and proactive aggression in adolescent boys. Aggress Behav.

[41] Goodman R (1997). The strengths and difficulties questionnaire: a research note. J Child Psychol Psychiatry.

[42] Arseneault L (2000). Mental disorders and violence in a total birth cohort: results from the Dunedin study. Arch Gen Psychiatry.

[43] Elliott DS, Huizinga DH, Klein MW (1989). Improving self-reported measures of delinquency. Cross-National Research in self-reported crime and delinquency.

[44] Grant VV (2009). Corrigendum to “psychometric evaluation of the five-factor modified drinking motives questionnaire—revised in undergraduates” [addictive behaviors 32/11 (2007) 2611–2632]. Addict Behav.

[45] Kessler RC (2002). Short screening scales to monitor population prevalences and trends in non-specific psychological distress. Psychol Med.

[46] Derogatis LR, BSI (1993). Administration, scoring and procedures manual for the brief symptom inventory.

[47] Stevens K (2009). Developing a descriptive system for a new preference-based measure of health-related quality of life for children. Qual Life Res.

[48] Telethon Kids Institute (2015). Young Minds Matter: the second Australian child and adolescent survey of mental health and wellbeing.

[49] Cornell D, Huang F (2016). Authoritative school climate and high school student risk behavior: a cross-sectional multi-level analysis of student self-reports. J Youth Adolesc.

[50] Cornell D (2012). Effects of validity screening items on adolescent survey data. Psychol Assess.

[51] Cornell DG, Lovegrove PJ, Baly MW (2014). Invalid survey response patterns among middle school students. Psychol Assess.

[52] Fitzmaurice GM, Laird NM, Ware JH (2011). Applied longitudinal analysis.

[53] Schafer JL, Graham JW (2002). Missing data: our view of the state of the art. Psychol Methods.

[54] Hox JJ (2010). Multilevel analysis techniques and applications.

[55] Clark DB, Winters KC (2002). Measuring risks and outcomes in substance use disorders prevention research. J Consult Clin Psychol.

[56] Conrod PJ, Castellanos N, Strang J (2010). Brief, personality-targeted coping skills interventions prolong survival as a non-drug user over a two-year period during adolescence. Arch Gen Psychiatry.

[57] Courtney RJ (2017). Predictors of retention in a randomised trial of smoking cessation in low-socioeconomic status Australian smokers. Addict Behav.

[58] Newton NC (2016). The long-term effectiveness of a selective, personality-targeted prevention program in reducing alcohol use and related harms: a cluster randomized controlled trial. J Child Psychol Psychiatry.

[59] Chinn S (2000). A simple method for converting an odds ratio to effect size for use in meta-analysis. Stat Med.

